# CEG: a database of essential gene clusters

**DOI:** 10.1186/1471-2164-14-769

**Published:** 2013-11-09

**Authors:** Yuan-Nong Ye, Zhi-Gang Hua, Jian Huang, Nini Rao, Feng-Biao Guo

**Affiliations:** Center of Bioinformatics and Key Laboratory for NeuroInformation of Ministry of Education, School of Life Science and Technology, University of Electronic Science and Technology of China, Chengdu, 610054 China

**Keywords:** CEG, Essential gene cluster, Antibacterial drug targets, Essential gene prediction

## Abstract

**Background:**

Essential genes are indispensable for the survival of living entities. They are the cornerstones of synthetic biology, and are potential candidate targets for antimicrobial and vaccine design.

**Description:**

Here we describe the Cluster of Essential Genes (CEG) database, which contains clusters of orthologous essential genes. Based on the size of a cluster, users can easily decide whether an essential gene is conserved in multiple bacterial species or is species-specific. It contains the similarity value of every essential gene cluster against human proteins or genes. The CEG_Match tool is based on the CEG database, and was developed for prediction of essential genes according to function. The database is available at http://cefg.uestc.edu.cn/ceg.

**Conclusions:**

Properties contained in the CEG database, such as cluster size, and the similarity of essential gene clusters against human proteins or genes, are very important for evolutionary research and drug design. An advantage of CEG is that it clusters essential genes based on function, and therefore decreases false positive results when predicting essential genes in comparison with using the similarity alignment method.

**Electronic supplementary material:**

The online version of this article (doi:10.1186/1471-2164-14-769) contains supplementary material, which is available to authorized users.

## Background

Essential genes are indispensable for the survival of living entities [1, 2], and the functions of proteins encoded by these genes are considered to be the foundation of life [1–3]. They are the cornerstones of synthetic biology [2, 4]. For example, in 2010, Venter *et al.* created a *Mycoplasma mycoides* cell based on the essential genes of *M. mycoides*[5]. Furthermore, analyses of the essential genes shared by different organisms further our understanding of the basic composition of cellular life [1, 6].

Since most antibiotic target gene products are involved in basic metabolic pathways, the essential genes of pathogens constitute attractive targets for antimicrobial drug and vaccine design [1–3, 7]. Hu *et al.*[8] and Roemer *et al*. [9] have respectively identified drug targets for *Aspergillus fumigatus* and *Candida albicans* based on corresponding essential genes identified from mouse models. Furthermore, Barh and Kumar [10], and Amineni *et al.*[11] successfully identified drug and vaccine targets *in silico* in *Neisseria gonorrhoeae* and *Leptospira interrogans* using the BLAST method against the Database of Essential Genes (DEG) [12, 13]. Identification and study of essential genes helps further the understanding of the origins of life and evolution, and can help determine the last universal common ancestor (LUCA) [6, 14].

Itaya *et al.* first investigated the essential genes of *Bacillus subtilis* on a large scale in 1995 [15]. Presently, essential genes have been determined at the genomic-scale in over 18 bacterial genomes. Zhang and colleagues [12, 13], and Chen *et al.*[16] have constructed two different databases, DEG and OGEE (Online GEne Essentiality database), respectively; these contain all published essential genes. The DEG [12, 13] is the first database of essential genes, and only collects genes determined by genome-wide experiments. Since its publication, the DEG database has been widely used in areas such as antibacterial drug target discovery and synthetic biology. OGEE [16] not only contains experimentally tested essential and non-essential genes, but also associates gene features such as expression profiles, duplication status, conservation across species, evolutionary origins, and involvement in embryonic development. It also stores text-mining data in addition to experimental data. Furthermore, the OGEE offers tools that allow users to compare gene essentiality among different gene groups, to compare the features of essential genes with non-essential genes, and for visualization of results. The CEG database differs from these databases in that it deposits essential genes in orthologous groups and not as single genes. The CEG_Match tool was developed for predicting essential genes in the CEG database based on function. CEG_Match significantly decreases false positive (FP) essential genes predictions in comparison with using direct sequence alignments.

## Construction and content

### Data acquisition

The original essentiality data for generating CEG were derived from DEG 6.5 [12, 13] (http://tubic.tju.edu.cn/deg/). After obtaining the data, we performed the following processes for each essential gene. First, as there are two groups of experimental data for *E. coli* in DEG, we removed any redundant data. For example, the gene GI:16128021 corresponds to three essentiality records in DEG, but is assigned to only one essentiality record in CEG. Second, genes were assigned to clusters based on their corresponding KEGG (Kyoto Encyclopedia of Genes and Genomes) Orthology (KO) [17] and Clusters of Orthologous Group (COG) category and function descriptions [18]. We investigated functional descriptions of all genes, or their KO identification, within each COG. If there were two or more different functional descriptions then the genes in a COG were separated into several smaller clusters. They were also separated based on KO. For example, genes within the code COG0008J are separated into three CEG clusters (CEG0151, CEG0208, and CEG0438), which correspond to the KOs k01885, k01886, and k09698. Data were manually curated at this step so that essential genes with synonymous functional descriptions but identical function were assigned within the same cluster. Consequently, resultant clusters contain only genes orthologous to each other, and will be regarded as one CEG cluster. Genes without COG identifications were assigned to the most probable COG identification and functional description through functional comparison and sequence alignment methodologies [19]. Third, each protein or gene in CEG is aligned against the whole human proteome or genome in the Human Protein Reference Database (HPRD), which contains 30046 human protein sequences [20, 21], using PSI-BLAST [22] or BLASTN tools [23]. The e-values of the best hits were recorded and named as ESAHP for proteins, and as ESAHG for genes. For each cluster, we give the e-value of the essential gene with the highest similarity as that of the cluster, and this provides a convenient resource for selecting targets of antibacterial drugs [2, 3, 7].

### Database design and implementation

The CEG database is executed using PHP scripts (http://php.net) on a Linux server, and queries a MySQL relational database (http://www.mysql.com). Its web interface is coded in PHP5 and HTML (http://www.w3.org).

**Figure 1:**
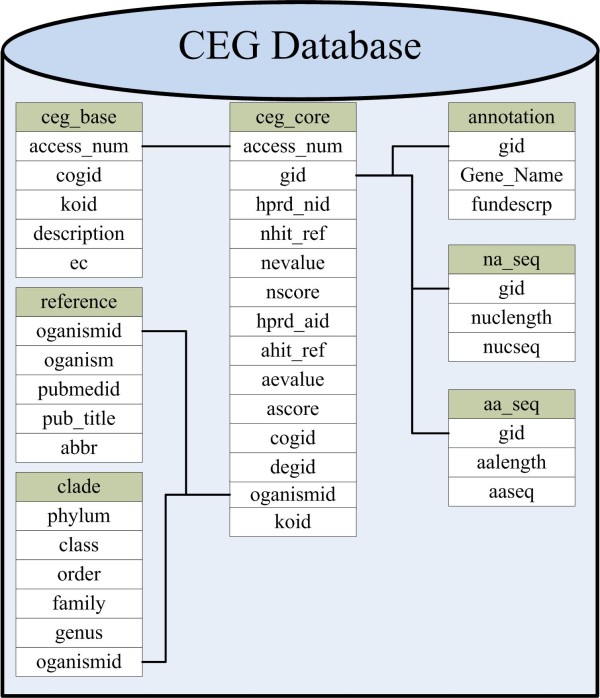
**Relationship among the tables used in the back-end of the CEG database.**

We created seven tables for the CEG database, and the relationship of these tables is presented in Figure 1. The “ceg_core” table is the core table of CEG, and contains the results of the best hit for each gene against the HPRD. The “ceg_base” table is helpful for integrating data from the “ceg_core” table to form CEG data (such as cluster size, ESAHG, ESAHP), and to produce dynamic new fields for the front-end of the database content.

### Database content

Each CEG cluster groups all orthologous genes that are essential for their bacterial hosts. This differs from DEG [12, 13] in that the CEG database stores essential genes in the form of orthologous gene groups rather than single genes. Viewing the size of the cluster allows users to decide whether an essential gene is conserved in multiple bacterial species or is species-specific. There are two different types of size number: one corresponding to the host number, and the other to the gene number. The gene number is not always identical to the host number because in some cases a genome may have multiple copies of the same gene. For example, the *Mycoplasma pulmonis* strain UAB CTIP has two *ligA* genes that belong to the cluster CEG_0001. The database also contains information on the highest similarity values for every essential gene or cluster against human genes or translated proteins. The data provided by CEG for each essential gene are very important for evolutionary research [6] and drug design [1, 2, 7].

**Figure 2:**
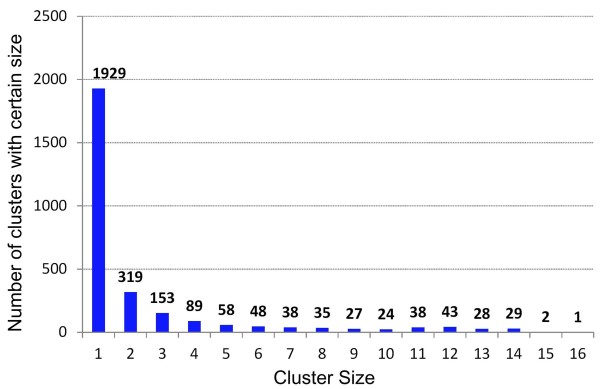
**The distribution of cluster sizes in the CEG database.**

In the current release, 6738 essential genes derived from DEG 6.5 are grouped into 2861 CEG clusters belonging to 16 prokaryotic strains which are listed in the reference page of the server (http://cefg.uestc.edu.cn/ceg/references.html). These essential genes were determined by different molecular techniques, such as single-gene knockouts, transposon mutagenesis, and RNA interference [12, 13]. Presently, the CEG database contains 932 clusters with two or more essential genes; it also contains 1929 pseudo clusters with only one essential gene. Of the 1929 pseudo clusters, 801 genes have neither KO nor COG identification numbers (id). Among the 932 genuine clusters, only four have neither KO nor COG identifications, and 809 have both KO and COG identifications. Detailed statistics of the classifications with KO and COG are given in Table 1. The cluster size (host number) in CEG ranges from 1 to 16 (Figure 2). Although CEG groups are functional-based, they are in high accordance with results from the sequence-based orthology assignment tool OrthoMCL [24] (June 2013 update). Of the 932 genuine clusters in CEG, 880 were found to be consistent with OrthoMCL groups following our comparison. For the non-consistent cases, most of the CEG clusters could be divided into two OrthoMCL groups.

**Table 1:** **KO and COG classifications in CEG**

Abbreviated strain name	No. of essential genes assigned within KO groups	No. of essential genes assigned within COG groups	No. of essential genes assigned within both KO and COG groups	% of essential genes assigned within KO groups.	% of essential genes assigned within COG groups	% of essential genes assigned within either of the two groups
aci	444	462	432	88.98	92.59	94.99
bsu	214	265	209	78.97	97.79	99.63
eco	564	647	558	79.21	90.87	91.71
ftn	331	289	279	84.44	73.72	86.99
hin	515	566	478	80.22	88.16	93.93
hpy	220	227	207	68.11	70.28	74.3
mge	282	303	270	74.02	79.53	82.68
mpu	262	270	252	84.52	87.1	90.32
mtu	461	535	452	75.08	87.13	88.6
pau	257	292	253	76.72	87.16	88.36
sao	304	334	302	86.61	95.16	95.73
spr	211	225	207	86.48	92.21	93.85
spu	286	302	286	94.7	100	100
stm	189	217	187	82.17	94.35	95.22
stt	338	343	337	95.75	97.17	97.45
vch	412	475	408	52.89	60.98	61.49

**Figure 3:**
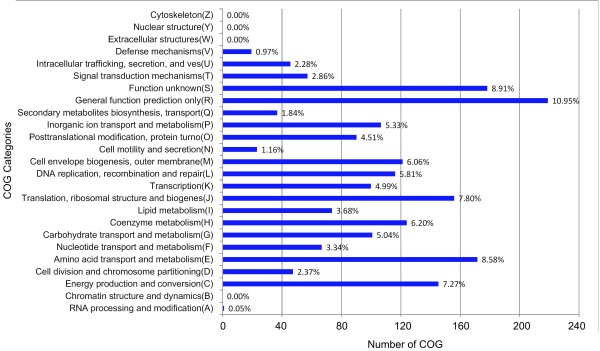
**The distribution of the COG functional categories of the CEG clusters.**

To comprehend the distribution of functions in the CEG clusters, the distribution of COG codes in CEG was analyzed. As shown in Figure 3, the top five functional categories of CEG were: R, S, E, J, and C. This suggests that functions involved in amino acid transport and metabolism, translation, ribosomal structure and biogenesis, and energy production and conversion have more essentiality in prokaryotic organisms. Further details on the database content can be found on the statistics page of the CEG database (http://cefg.cn/ceg/statistics.html). Similarity alignment results for every cluster or gene against human proteins are given in CEG. For each gene, the e-value of 10e-3 against human genome is usually regarded as the threshold for choosing target genes or proteins for antibacterial drugs to avoid possible toxicity to humans [2, 7]. Consequently, 3900 essential genes were found not to be conserved in the human genome according to this value, and could constitute potential targets for innocuous antibacterial drugs [1, 2, 7]. Generally, there are three rough criteria rules to be considered when choosing an essential cellular function as an antibacterial drug target [7]: Rule 1) the highly conserved function in a range of pathogens and the conserved level (sequence or function) in different pathogens; Rule 2) essentiality of the gene for the bacteria; and Rule 3) having no highly conserved function in humans. Our database provides information for each of these criteria for each essential gene. Therefore, it will be helpful for antimicrobial drug design.

We developed the tool CEG_Match for prediction of essential genes based on whose functions. The ideology for this method is as described by Guo *et al*. [25]. When using the CEG_Match to predict essential genes, the end-user only needs to provide standard names or synonymous names of genes in the query bacterial genome. For example, ‘*dnaA*’ is the name of a gene encoding one type of chromosomal replication initiation protein. This information is usually contained in files with the extension ptt in GenBank and RefSeq annotations. Note that the CEG_Match is aware of gene name synonyms.

## Utility

### User interface and database usage

We have created a freely accessible web interface for visiting the CEG database. It includes five core-page sections: “home”, “browse”, “search”, “blast”, and “predict”.

### Home page

Users can access the CEG database at the URL http://cefg.uestc.edu.cn/ceg/. The home page contains an introduction to the CEG database and provides a contact email address for users to leave feedback suggestions to the administrators. A brief user guide is also provided on this page.

### Browse pages

This section includes overview page, cluster information page, and gene information page. In the overview page, users can browse the basic information of each gene cluster (or gene) in the CEG database. This information includes CEG (or gene) id, cluster name, KO id, COG id, cluster size, enzyme id (EC), the number of strains covered, and the e-value of the similarity alignment for every cluster (or gene) compared against human proteins [21]. The cluster information page provides the phyletic profile of a cluster, and a list of included genes. Users are easily able to estimate the conservation of a cluster at different clade levels (phylum, class, order, family, and genus). Links to external information on each essential gene or cluster are also given. For example, users are able to open an information page for a group in the KO website [17] through links on the KO id, and quickly investigate whether an equivalent gene appears in a CEG cluster. For clusters that do not have COG codes in the COG database, or a KO id in KEGG, an exception-handling interface alerts the end-user that these records are not found in the KO database. In addition, a link to retrieve the original information in DEG [12, 13] is provided for each gene. Clicking the HPRD id [21] opens a page containing information on the best HPRD hit in the human genome. The whole essential gene set in the CEG database can be sorted in ascending or descending order according to indexes such as cluster size and similarity value. This facilitates users mine information of interest. For example, if a researcher wanted to design a broad-spectrum antibiotic, they would quickly be able to identify that the *dnaA* gene has potential as a drug target because of it being conserved in 13 strains, and not being homologous to human genes. Meanwhile, the *gltD* gene would quickly be dismissed as a drug target because it is only conserved in two strains, and is homologous to human genes.

### Search & blast pages

End-users can search the CEG database by cluster name, cluster size, cluster function, or cluster id. CEG allows users to paste or upload sequences, and BLAST the query sequences against all clusters of essential genes contained within the database.

**Figure 4:**
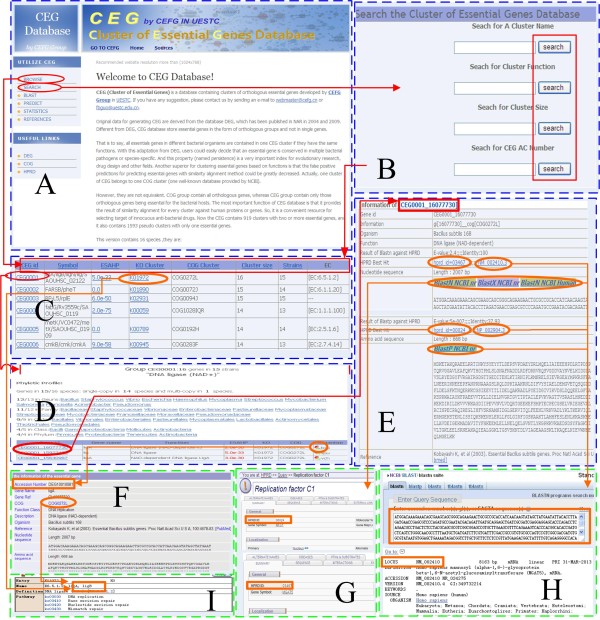
**The relationship of internal and external pages, and an example of using the CEG database. A**, home page; **B**, search page; **C**, **D**, and **E**, browse pages. **A** to **E** are the internal pages of the CEG database, and these are surrounded by a blue dashed line. **F** to **I** are external pages, and are surrounded by a green dashed line. **F**, DEG database; **G**, HPRD database; **H**, NCBI database; **I**, KO database.

The relationship of the website pages, and an example of how to use the CEG database, is given in Figure 4. In addition, a download page is provided for users to download CEG data (http://cefg.cn/ceg/sources.php). The CEG database interfaces have been tested using Internet Explorer, Epiphany, Iceweasel, Mozilla Firefox, Google Chrome, Opera, and Safari on the Windows XP/Vista/7/8, Linux and Mac operating systems.

### CEG_Match usage

**Figure 5:**
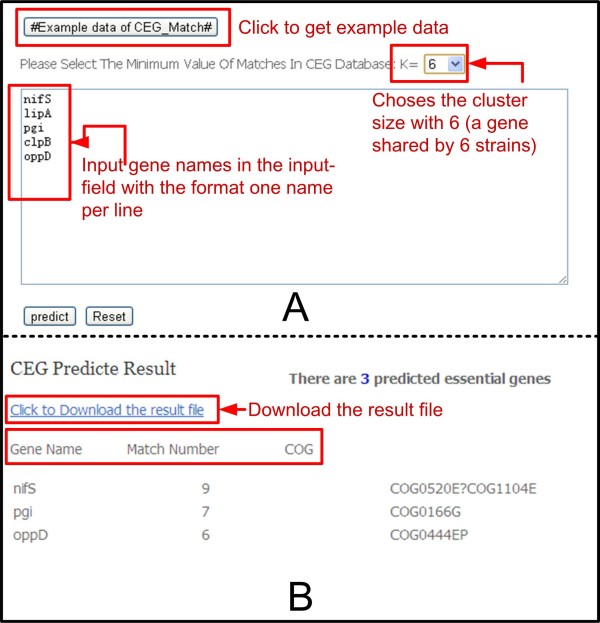
**The example of using CEG_Match described in the text. A**: input page; **B**: results page.

CEG_Match was developed for predicting essential genes from the CEG database according to their functions. Users can access this tool at the following URL: http://cefg.uestc.edu.cn/ceg/predict.php. Users are required to input gene names into the input-field in a one name per line format. They are also required to select the cluster size (number of strains covered, K) before running the tool. An example of how to use CEG_Match is given in Figure 5. The result page contains information on the gene name, cluster size, and COG id of the essential genes. The results can be downloaded as a text-file from the result page. The use of CEG_Match greatly decreases FPs when predicting essential genes in comparison with using the similarity alignment method [1–3, 7].

### CEG usage case

*Campylobacter jejuni* is a zoonosis pathogenic bacterium. It can cause a variety of human and animal diseases. It is considered the main cause of bacterial diarrhea in humans [26]. The complete genome sequence of *C. jejuni* NCTC 11168 was sequenced in 2012 [27]. We download these data from the NCBI ftp site. The genome has 1621 proteins (or genes), 803 of which have been annotated with detailed functions (with standard gene names given in the annotation file). In the following example, we predicted essential genes of *C. jejuni* NCTC 11168 using CEG_Match, and identified potential drug target genes *in silico*.

First, we collected the 803 annotated gene names, and predicted 374 genes as essential using a setting of K = 3 in CEG_Match (Additional file 1). Second, we retrieved the ESAHP values of these genes from the CEG database. Genes with an ESAHP value larger than 10e-3 were considered as potential targets of innocuous antibacterial drugs, and this led to 120 potential drug targets being predicted in *C. jejuni* NCTC 11168 (Additional file 1: Table S1). Fifty-seven genes, with a match number greater than seven in the CEG database, have a high potential to be broad-spectrum antibiotic drug targets (Additional file 1: Table S1).

## Discussion

### DEG, OGEE *vs.* CEG

Bacterial essential genes are the subject of increased recent attention because of their importance in the fields of antibacterial drug design [1–3, 7], synthetic biology [2, 4], and life origin research [1, 6]. The essential gene databases, DEG [12, 13] and OGEE [16], contain essential genes determined by experimental methods, and have been used for evolutionary research [6] and drug design [7]. However, they only include basic sequence information rather than a full integration of resources that are convenient for evolutionary research and drug design [7]. The CEG database was developed with the rationale of enriching the information contained in the existing essential gene databases. In the CEG database, each cluster (gene) is provided with indices such as cluster size, and the results of similarity alignments for every cluster (gene) against human proteins. This information is not found in DEG or OGEE. The cluster size is helpful when devising broad-spectrum or specific drugs (corresponding to Rule 1 mentioned above). It is also useful for functional or evolutionary genomic bacteria research.

**Figure 6:**
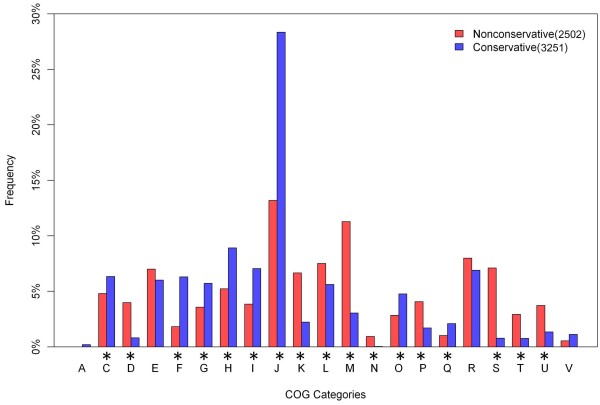
**Different COG functional category distributions between groups conserved and non-conserved in humans.** The * symbol signifies that the category has a significant difference between the two groups (Z-test, *P* < 0.01).

3900 essential genes were not conserved in the human genome when using an e-value of 10e-3, and these constitute potential targets for innocuous antibacterial drugs (corresponding to the Rule 3) [1, 2, 7]. To clarify whether this conservation to humans is related to functional category, we divided all essential genes into two groups, based on the e-value of 10e-3, for further analysis. The results of this analysis of COG categories are given in Figure 6. The first group of essential genes, belonging to the COG categories C, F, G, H, I, J, O, and Q, largely function in transportation and metabolism, are more abundant in groups conserved to human proteins. The COG codes D, K, L, M, N, P, T, and U appear more often in the second group. This suggests that the design of antibacterial drugs should be aimed at interrupting cell division, transcription, DNA replication, recombination and repair, cell outer membrane and envelope biogenesis, and secretion functions of pathogenic bacteria, but should avoid transportation and metabolism functions.

### Sequence homology approaches *vs.* CEG_Match

Sequence homology approaches are the foundation for functional inference. Although powerful, homology approaches have their limitations. For instance, they do not give information on direct functional links among non-homologous genes [28]. Lord *et al*. found a strong correlation between gene annotation (function) and sequence similarity (homology); however, some protein pairs deviate from this trend [29]. A consideration of the above factors led to the development of the CEG_Match tool for predicting essential genes contained in the CEG database. This tool and methodology make full use of the annotations of the genes. Because the annotated functions are mainly obtained through homology alignment, it follows that the tool is also based on alignment data. However, it does not directly use sequence alignments, but makes use of alignment-generating-annotation information.

**Figure 7:**
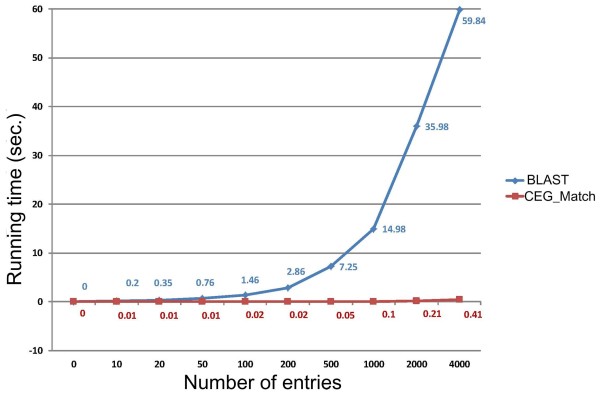
**Comparison of CEG_Match and BLAST running speeds.**

We compared the predicted results and running speeds of BLAST and CEG_Match. According to the method of Tian and Skolnick [30], we chose identity >40% and e-value < 1e-10, and identity >50% and e-value <1e-10, for BLAST predicting. In our case, these parameters generated the best result for BLAST alignment. We found the number of essential genes predicted by CEG_Match to be closest to the number observed when the parameter of CEG_Match was set as K = 3 or K = 4. Therefore a cluster size > =3, or > =4 (K = 3, or K = 4) is recommended to reduce the prediction of FPs, and improve the accuracy of the prediction. The predicted results between BLAST and CEG_Match are given in Table 2. To reveal the evolutionary relationship between organisms in the database and the investigated species, we defined an index named minimum distance (MD). This is the value of the minimum phylogenetic distance between the organisms in the database and the investigated strains measured by CVTree [31, 32]. Use of BLAST revealed that the accuracy and gene loss rate were significantly correlated with MD ((Pearson's correlation coefficient: *r* = –0.579, *P* = 0.018; and *r* = 0.666, *P* = 0.005, respectively). However, the correlations were not significant at *P* = 0.05 when using the CEG_Match method. This implies that the prediction effect of sequence homology approaches depends on the distance in the database, and is instable. The method of CEG_Match has a constant prediction effect, and is more independent of phylogenetic distance. Moreover, correlations between FPs and minimum distance were not found with either of the two methods ((Pearson's correlation coefficient: BLAST: *r* = –0.332, *P* = 0.209; CEG_Match: *r* = –0.210, *P* = 0.435). We propose a new measure, FP’ = FP _(CEG_Match, K=4)_–FP _(BLAST, identity >50%)_. This reveals significant differences in FP’ (Wilcoxon test, *P* = 0.040) between the two groups when separated on distance. This means that the comparison of gene functions (or names) by CEG_Match will have a much higher accuracy and lower FPs than direct sequence alignment when the query genome is well annotated but is not closely related to any strains in the database. To compare the running speeds of CEG_Match and BLAST, we measured the running time of different number of entries (Figure 7). This revealed that CEG_Match runs faster than BLAST.

**Table 2:** **The predicted results between blast and CEG_match**

Abbreviated strain name	BLAST (e-value <1e-10)						CEG_Match						Min. distance
	Identity >50			Identity >40			K = 3			K = 4			
	Accuracy (%)	False positive rate (%)	Loss rate (%)	Accuracy (%)	False positive rate (%)	Loss rate (%)	Accuracy (%)	False positive rate (%)	Loss rate (%)	Accuracy (%)	False positive rate (%)	Loss rate (%)	
aci	69.9	42.9	54.1	75.2	53.7	36.5	79.6	28	36.5	77.1	23.7	42.7	0.488
bsu	79.1	60.1	35.1	83.1	71.9	19.6	84	44.1	28	82.5	35.3	32.5	0.487
eco	69.4	56.9	46.6	67.7	65.5	43.1	71.2	40	50.8	70.1	30.6	55.8	0.326
ftn	67.3	37.5	57.9	72.2	48.2	38.8	78.1	36.5	32.9	78.2	28.9	36	0.493
hin	53.8	55.4	64.6	54.1	56.3	55.8	59	45.7	61.7	58.7	43.6	65.9	0.477
hpy	52.8	66	88.9	56.5	68.6	74.6	57.5	60.6	75.2	56.9	59.8	77.7	0.497
mge	53.8	6.8	89.2	62	6.1	67.5	71.3	6.9	39.4	70.4	5.9	45.4	0.496
mpu	56.8	19.4	83.9	70.3	16.7	53.2	64.5	16.3	66.8	64.7	10.8	68.1	0.496
mtu	56	41.8	86.2	62.7	49.5	69.1	71.4	30	53.6	68.3	28.2	60.6	0.497
pau	72.2	72.2	47.5	75	79.2	35.2	79.2	57.8	36.4	77	53.1	42.1	0.488
sao	75.3	50.2	41.3	78.6	57.5	29.6	74.2	20.7	49.9	74.5	14.6	51.9	0.074
spr	51.9	85.2	84.4	55.5	84	70.9	69.6	52.3	54.1	68.5	52.4	59	0.493
spu	76.1	57	36.8	79.6	62.4	24.2	83	42.4	26.8	80.6	38.2	33.4	0.074
stm	67.4	87.6	43.5	66.7	89.3	38.7	72	79.8	43.9	71.6	75.6	47.8	0.11
stt	85.7	71	9.1	83	76.1	8.2	89.8	49.2	13	87.8	36.9	20.4	0.11
vch	60	61.9	65.7	59.3	67.7	60.2	66.9	26.7	62.6	65.7	23.6	66	0.477

### Further developments

The CEG database will be updated when new bacterial essential genes are experimentally determined at the genome scale. In next version, we will also take OGEE as one data source. Furthermore, phylogenetic relationships among genes in every cluster and the Gene Ontology information of CEG genes will be incorporated into the CEG database. To make the CEG database more comprehensive for drug design and related fields, the protein structures of each essential gene will also be incorporated into the database.

## Conclusions

In this study, we propose a terminology called Cluster of Essential Genes (CEG), and construct a database to deposit these essential gene clusters. The CEG database has the following features: (I) it stores essential genes in the form of orthologous groups instead of as single genes; (II) it provides an essential gene prediction tool (CEG_Match), which could greatly decrease the number of FPs when predicting essential genes in comparison to the similarity alignment method; (III) it makes it easy for the end-user to determine whether an essential gene is conserved in multiple species or is species-specific; and (IV) ESAHP and ESAHG values in the CEG database allow the end-user to easily obtain the similarity of every cluster against human proteins or genes. Features (III) and (IV) are important properties for drug target discovery [1–3, 7].

## Availability and requirements

The CEG database is publicly available at http://cefg.uestc.edu.cn/ceg and http://cefg.cn/ceg.

## Electronic supplementary material

Additional file 1: Table S1: List of predicted essential genes and antibiotic drug targets. (XLSX 53 KB)

## Abbreviations

FP: False positive
DEG: Database of essential genes
COG: Clusters of orthologous groups
HPRD: The human protein reference database
OGEE: Online gene essentiality database
ESAHP: The e-value of similarity alignment (PSI-BLAST) for every cluster against human proteins
ESAHG: The e-value of similarity alignment (BLASTN) for every cluster against human genes
aci: *Acinetobacter baylyi*
bsu: *Bacillus subtilis* 168
eco: *Escherichia coli*
ftn: *Francisella novicida* U112
hin: *Haemophilus influenzae*
hpy: *Helicobacter pylori*
mtu: *Mycobacterium tuberculosis*
mge: *Mycoplasma genitalium*
mpu: *Mycoplasma pulmonis* UAB CTIP
pau: *Pseudomonas aeruginosa*
stt: *Salmonella enterica* serovar Typhi
stm: *Salmonella typhimurium*
spu: *Staphylococcus aureus* N315
sao: *Staphylococcus aureus* NCTC 8325
spr: *Streptococcus pneumoniae*
vch: *Vibrio cholera* N16961.
